# Using Blood Indexes to Predict Overweight Statuses: An Extreme Learning Machine-Based Approach

**DOI:** 10.1371/journal.pone.0143003

**Published:** 2015-11-23

**Authors:** Huiling Chen, Bo Yang, Dayou Liu, Wenbin Liu, Yanlong Liu, Xiuhua Zhang, Lufeng Hu

**Affiliations:** 1 College of Physics and Electronic Information Engineering, Wenzhou University, Wenzhou, China; 2 College of Computer Science and Technology, Jilin University, Changchun, China; 3 Key Laboratory of Symbolic Computation and Knowledge Engineering of Ministry of Education, Jilin University, Changchun, China; 4 College of Pharmaceutical Sciences, Wenzhou Medical University, Wenzhou, China; 5 Pharmaceutical Department, The First Affiliated Hospital of Wenzhou Medical University, Wenzhou, China; Wayne State University, UNITED STATES

## Abstract

The number of the overweight people continues to rise across the world. Studies have shown that being overweight can increase health risks, such as high blood pressure, diabetes mellitus, coronary heart disease, and certain forms of cancer. Therefore, identifying the overweight status in people is critical to prevent and decrease health risks. This study explores a new technique that uses blood and biochemical measurements to recognize the overweight condition. A new machine learning technique, an extreme learning machine, was developed to accurately detect the overweight status from a pool of 225 overweight and 251 healthy subjects. The group included 179 males and 297 females. The detection method was rigorously evaluated against the real-life dataset for accuracy, sensitivity, specificity, and AUC (area under the receiver operating characteristic (ROC) curve) criterion. Additionally, the feature selection was investigated to identify correlating factors for the overweight status. The results demonstrate that there are significant differences in blood and biochemical indexes between healthy and overweight people (p-value < 0.01). According to the feature selection, the most important correlated indexes are creatinine, hemoglobin, hematokrit, uric Acid, red blood cells, high density lipoprotein, alanine transaminase, triglyceride, and γ-glutamyl transpeptidase. These are consistent with the results of Spearman test analysis. The proposed method holds promise as a new, accurate method for identifying the overweight status in subjects.

## Introduction

The overweight condition is a rapidly growing health concern in developed and developing countries. The number of overweight and obese people has continuously increased across the world. According to a 2014 WHO (World Health Organization) survey, 39% of adults were overweight. In China, the number of overweight people in the 7–18 year-old age range increased from 1.8 to 11.9% between 1985 and 2005 [1]. Being overweight raises the risk for a number of diseases, such as high blood pressure, blood glucose, dyslipidemia, diabetes mellitus, coronary heart disease, certain forms of cancer, stroke, and sleep-breathing disorders [2–5]. Consequently, being able to identify the overweight status is an important public health concern.

To date, several machine learning methods have been proposed to automatically diagnose subjects’ body mass index (BMI). Most have exhibited promising accuracy. Hardalac et al. [6] developed neuro-fuzzy systems that included NEFCLASS and CANFIS; they analyzed medical parameters such as subjects’ BMIs and divergent arteries. The results showed that the classification performance of NEFCLASS was better than that of CANFIS, and that obesity affects BMI rather than divergent arteries. Zhang et al. [7] proposed using logistic regression and six data mining methods to identify overweight versus obese children at different ages using the parameters of children from birth to around 3 years old. The results showed that the incorporation of non-linear interactions enhanced classification performance, and data mining methods such as artificial neural networks (ANN) and Bayesian methods perform better than logistic regression. Ergun [8] proposed the ANN and logistic regression-based automatic diagnosis systems for identifying obese from non-obese subjects using artery parameters and BMI. The results demonstrated that the classification performance of ANN was better than logistic regression, and BMI is more affected than divergent arteries in obese subjects. Valavanis et al. [9] developed hybrid ANN-based methods, including PDM-ANN and GA-ANN, for identifying overweight versus healthy subjects using gender, genes, and nutrition habits as parameters. These methods showed promising generalization ability; important differentiating factors were identified, including gender, six genetic variations, and 18 nutrition-related variables. Heydari et al. [10] proposed ANN and logistic regression for classifying normal and obese classes using subjects’ demographics, lifestyle information, and anthropometric measurements. The results showed that both ANN and logistic regression are effective classifiers for obesity detection, and they are not significantly different in capabilities. Recently, Lee et al. [11] predicted BMI status (normal, overweight, and obese) by logistic regression, bagging and random forest algorithms. Based on the combination of voice features, the results showed that the classification models built by logistic regression were better than the other two algorithms.

As can be seen from these studies, ANN is a popular method for detecting subjects’ BMI statuses; it achieves promising classification accuracy among the available machine learning methods. However, traditional ANN training algorithms such as the back propagation method can become trapped in local minima because they are gradient descent-based algorithms. Another disadvantage of ANN is that there are many network parameters that need to be carefully specified. Unlike ANN, the extreme learning machine (ELM) [12] chooses input weights and hidden biases randomly, and output weights are analytically determined using the Moore-Penrose generalized inverse. As a result, the ELM learns more quickly and keeps fewer fine- tuning parameters than ANN, while maintaining excellent generalization performance. ELM has been applied in fields such as disease diagnosis [13, 14], image quality assessment [15], face recognition [16], land cover classification [17] and hyperspectral images classification [18]. To the best of the authors’ knowledge, ELM has not yet been used in overweight modeling applications.

In addition, related works on overweight detection reveal that there is no scientific literature that explores overweight modeling using blood samples. Blood examination is standard in the medical field, used to evaluate liver function, renal function, and blood-lipid and glucose levels. This study is the first to explore the potential of ELM in overweight modeling using information from blood samples. Moreover, this study is the first attempt to identify the most informative and influential factors that discriminate overweight from healthy subjects. For comparison purposes, ANN, the most widely used model in overweight modeling and the advanced support vector machines [19] (SVM), were also used for overweight modeling. In addition, feature selection was employed to identify correlating factors. The Fisher Score selection was employed for pre-processing before the classification models were constructed. The classification accuracy (ACC), AUC, sensitivity, and specificity of the proposed approach were examined by comparing the results against the real-life dataset. Samples were collected from The First Affiliated Hospital of Wenzhou Medical University. Promisingly, the developed ELM-based approach achieved 90.54% ACC, 90.17% AUC, 83.54% sensitivity, and 96.80% specificity.

In summary, this paper makes the following contributions: (1) It proposes a new perspective for overweight modeling using blood samples; (2) It proposes an effective and efficient approach for automatically identifying the overweight status; and (3) The most relevant indexes were incrementally detected with the aid of the feature selection.

## Materials

### 1. Ethics Statement

The Medical Ethics Committee of The First Affiliated Hospital of Wenzhou Medical University approved of the study, which was conducted in accordance with the Declaration of Helsinki. As a retrospective investigation, no written consents were obtained from participants, and all individual participant information was collected anonymous. Samples and participant data were provided by the information department of The First Affiliated Hospital of Wenzhou Medical University. Participants with significant cardiovascular, hepatic disease, renal disease, uncontrolled thyroid disease, significant central nervous system-related, or psychiatric disorders were excluded from this study. The ethics committees waived the need for written informed consent and approved this consent procedure.

### 2. Data Preparation

A stadiometer was used to measure height, and a self-zeroing weight scale was used to measure weight. Participants wore foam slippers, and a paper shirt and pants during the weighing process. There were 179 male and 297 female participants in this study. According to the WHO definition, participants were included in this study if their BMI was 25.0–30 kg/m^2^, and defined as overweight [20]. Samples were divided into healthy people (n = 251) and overweight people (n = 225); their BMIs were 21.79 ± 0.92 kg/m^2^ and 26.17 ± 0.84 kg/m^2^, respectively. Fasting venous blood samples of all participants were collected into separation gel tubes, and blood and biochemical indexes were determined using a Hitachi 705/717 instrument at the physical examination center of the hospital. A total of 18 blood indexes and 16 biochemical indexes were included in this study; Table 1 includes their detailed descriptions, as well as age, red blood cell count, and white blood cell in urine. All these data have been uploaded as an attachment in supplementary file which can be accessed freely.

**Table 1:** All of the features used in this study and their brief descriptions.

Feature	Brief description	Feature	Brief description
**X**_**1**_	Age	**X**_**20**_	absolute value of monocyte (AVM)
**X**_**2**_	triglyceride (TG)	**X**_**21**_	red blood cell (RBC)
**X**_**3**_	glucose (GLU)	**X**_**22**_	red blood cell in urine (RBCU)
**X**_**4**_	low density lipoprotein (LDL)	**X**_**23**_	hematokrit (HCT)
**X**_**5**_	high density lipoprotein (HDL)	**X**_**24**_	percentage of leukomonocyte (PLC)
**X**_**6**_	total cholesterol (CHO)	**X**_**25**_	absolute value of leukomonocyte (AVLC)
**X**_**7**_	alanine transaminase (ALT)	**X**_**26**_	mean corpuscular volume (MCV)
**X**_**8**_	aspartate aminotransferase (AST)	**X**_**27**_	mean corpuscular hemoglobin (MCH)
**X**_**9**_	γ-glutamyl transpeptidase (γ-GT)	**X**_**28**_	mean corpuscular hemoglobin concentration (MCHC)
**X**_**10**_	total protein (TP)	**X**_**29**_	mean platelet volume (MPL)
**X**_**11**_	albumin (ALB)	**X**_**30**_	absolute value of eosinophils (AVE)
**X**_**12**_	creatinine (CR)	**X**_**31**_	percentage of eosinophils (PE)
**X**_**13**_	urea nitrogen (BUN)	**X**_**32**_	hemoglobin (HB)
**X**_**14**_	alkaline phosphatase (AKP)	**X**_**33**_	blood platelet (PLT)
**X**_**15**_	total bilirubin (TBIL)	**X**_**34**_	thrombocytocrit (THR)
**X**_**16**_	direct bilirubin (DBIL)	**X**_**35**_	percentage of neutrophils (PN)
**X**_**17**_	uric acid (UA)	**X**_**36**_	absolute value of neutrophils (AVN)
**X**_**18**_	white blood cell in urine (WBCU)	**X**_**37**_	red cell volume distribution width (RBCVD)
**X**_**19**_	percentage of monocyte (PMC)		

Statistical analysis was performed using SPSS 17 software. The BMI, age, blood, and biochemical indexes of the two groups were analyzed by a One-Way ANOVA test to discover statistical differences. Table 2 lists detailed statistical descriptions. The correlation between BMI with blood and biochemical indexes were analyzed using a Spearman test. The p-values that were less than 0.05 (the 5% significance level) were considered to indicate statistical significance in all analyses.

**Table 2:** Characteristics of blood and biochemical parameters in healthy and overweight people.

Index	Overweight (n = 225)				Healthy (n = 251)				
	Mean	SD	Min	Max	Mean	SD	Min	Max	p-value
AGE	44.73	10.41	22.00	76.00	42.90	10.41	24	82	0.057
TG	2.20	1.80	0.46	14.17	0.98	0.64	0.23	5.73	0.000
GLU	5.92	1.24	4.50	17.00	5.44	0.88	4.20	15.20	0.000
LDL	2.87	0.71	1.28	5.85	2.57	0.76	0.76	5.82	0.000
HDL	1.23	0.28	0.69	2.21	1.61	0.32	0.88	2.72	0.000
CHO	5.00	0.91	2.98	9.97	4.69	0.90	2.54	8.25	0.000
ALT	35.66	23.31	4.00	137.00	16.46	10.01	2.00	69.00	0.000
AST	25.13	9.84	10.00	83.00	19.37	5.91	10.00	49.00	0.000
γ-GT	56.95	65.98	10.00	564.00	15.95	10.57	6.00	121.00	0.000
TP	76.65	3.50	66.00	86.60	76.14	3.80	66.20	85.50	0.130
ALB	47.69	2.66	38.80	54.50	46.48	2.38	40.80	53.30	0.000
CR	72.46	13.41	41.00	107.00	51.92	7.01	34.00	78.00	0.000
BUN	5.28	1.22	2.80	9.10	4.73	1.17	2.30	8.50	0.000
AKP	82.71	20.65	32.00	170.00	65.35	19.91	23.00	185.00	0.000
TBIL	11.82	4.13	5.00	27.00	9.82	3.20	5.00	22.00	0.000
DBIL	3.76	1.45	2.00	11.00	3.10	1.06	2.00	7.00	0.000
UA	381.48	83.29	98.00	601.00	267.31	50.55	128.00	403.00	0.000
WBCU	11.27	25.04	0.00	203.10	31.67	56.41	0.30	474.50	0.000
PMC	0.07	0.02	0.02	0.14	0.07	0.02	0.03	0.14	0.005
AVM	0.50	0.17	0.00	1.30	0.39	0.12	0.20	0.80	0.000
RBC	5.04	0.48	3.75	6.87	4.46	0.38	3.45	6.03	0.000
RBCU	54.01	473.50	0.00	6977.40	42.96	116.47	1.20	1500.00	0.721
HCT	0.46	0.04	0.31	0.53	0.41	0.03	0.29	0.46	0.000
PLC	0.37	0.07	0.15	0.59	0.37	0.08	0.11	0.60	0.781
AVLC	2.47	0.70	0.00	5.40	2.11	0.57	0.90	4.60	0.000
MCV	91.04	6.23	62.20	108.20	91.22	6.45	56.50	104.30	0.757
MCH	30.17	2.53	18.90	34.80	29.40	2.48	15.40	33.10	0.001
MCHC	331.01	11.65	268.00	357.00	321.98	9.66	272.00	345.00	0.000
MPL	10.83	1.91	0.00	14.10	10.71	2.17	0.00	14.20	0.521
AVE	0.17	0.12	0.00	0.60	0.13	0.10	0.00	0.90	0.000
PE	0.02	0.02	0.00	0.10	0.02	0.01	0.00	0.11	0.011
HB	151.59	15.03	83.00	179.00	130.51	9.63	78.00	149.00	0.000
PLT	223.42	49.68	102.00	437.00	226.57	51.96	85.00	451.00	0.500
THR	0.24	0.06	0.00	0.45	0.24	0.07	0.00	0.42	0.772
PN	0.53	0.08	0.34	0.78	0.54	0.08	0.30	0.84	0.403
AVN	3.67	1.25	0.00	9.60	3.16	1.04	1.30	7.30	0.000
RBCVD	12.85	0.98	11.40	19.30	12.90	1.10	11.60	20.90	0.536

## Methods

### 1. Fisher Score

The Fisher Score [21] is one of the most commonly used and efficient supervised feature weighing methods. It determines the most discriminative features according to the fisher criterion. Given the dataset of n instances {*x*_*i*_, *y*_*i*_}, where $x_{i}\in R^{m}$ represents that the input feature space has *m* features and *y*_*i*_ ∈ {1, 2,…, *c*} is the corresponding class labels. The score of the *m*-th feature can be directly measured as follows:

$$F_{m}=\frac{\sum_{i=1}^{c}n_{i}{\left(\mu_{i}^{m}-\mu^{m}\right)}^{2}}{{\left(\sigma_{}^{m}\right)}^{2}}.$$ (1)

where ${\left(\sigma^{m}\right)}^{2}=\sum_{i=1}^{c}n_{i}{\left(\sigma_{i}^{m}\right)}^{2}$, *n*_*i*_ denotes the number of instances in class *i*, $\mu_{i}^{m}$ and *μ* ^*m*^ represent the mean value of class *i* and the global mean value corresponding the *m*-th feature, respectively. $\sigma_{i}^{m}$ and *σ* ^*m*^ mean the variance of class *i* and the global variance corresponding the *m*-th feature, respectively. According to Eq (1), the larger score value represents that the *m*-th feature has more discriminative power between the different classes.

### 2. Extreme Learning Machine (ELM)

This section provides a brief description of ELM; refer to [12, 22] for more information. Given a training dataset with *N* samples $R={\left\{x_{i},t_{i}\right\}}_{i=1}^{N}$, *x*_*i*_ ∈ *R* ^*n*^ is the input feature vector with *n* features, and *t*_*i*_ ∈ *R* ^*m*^ represents the target vector with *m* dimensions. The output of ELM can be written as follows [12]:

$$\sum_{i=1}^{k}\beta_{i}g(w_{i}\cdot x_{j}+b_{i})=o_{j},\;j=1,2,\ldots ,N$$ (2)

where *g*(*x*) is the activation function, *k* is the number of hidden neurons, *β*_*i*_ is the weight vector between the *i*th hidden neuron and the output layer, *w*_*i*_ is the weight vector between the *i*th neuron in the hidden layer and the input layer, and *b*_*i*_ indicates the bias of the *i*th neuron in the hidden layer, *o*_*j*_ is the target vector of the *j*th input data. If ELM can approximate these *N* samples with zero error, we can get $\sum_{i=1}^{k}\beta_{i}g(w_{i}\cdot x_{j}+b_{i})=t_{j},\;j=1,2,\ldots ,N$. The above equation can be reformulated as follows:

Hβ=T (3)

where H [23] represents the hidden layer output matrix of the neural network:

$$\text{H}={\left(\begin{array}{ccc} g(w_{1}\cdot x_{1}+b_{1}) & \ldots & g(w_{k}\cdot x_{1}+b_{k})\\ \vdots & \ddots & \vdots \\ g(w_{1}\cdot x_{N}+b_{1}) & \cdots & g(w_{k}\cdot x_{N}+b_{k}) \end{array}\right)}_{N\times k}$$ (4)

*β* = [*β*_1_,⋯, *β*_*k*_]^*T*^ is the matrix of output weights from the hidden layer to the output layer, and T = [*t*_1_,⋯, *t*_*N*_]^*T*^ denotes target labels’ vectors. Under the assumption that [24, 25] the input weights and the hidden layer biases of single hidden layer feed-forward neural network (SLFNs) can be arbitrarily given, the output weights *β* can be analytically determined by the Moor-Penrose (MP) generalized inverse of matrix H, as is shown in the following Equation:

$$\beta ={\text{H}}^{†}\text{T}$$ (5)

By using the MP inverse method, the ELM generalization performance can be achieved with a dramatically increased learning speed [22].

### 3. The Proposed Method

**Fig 1:**
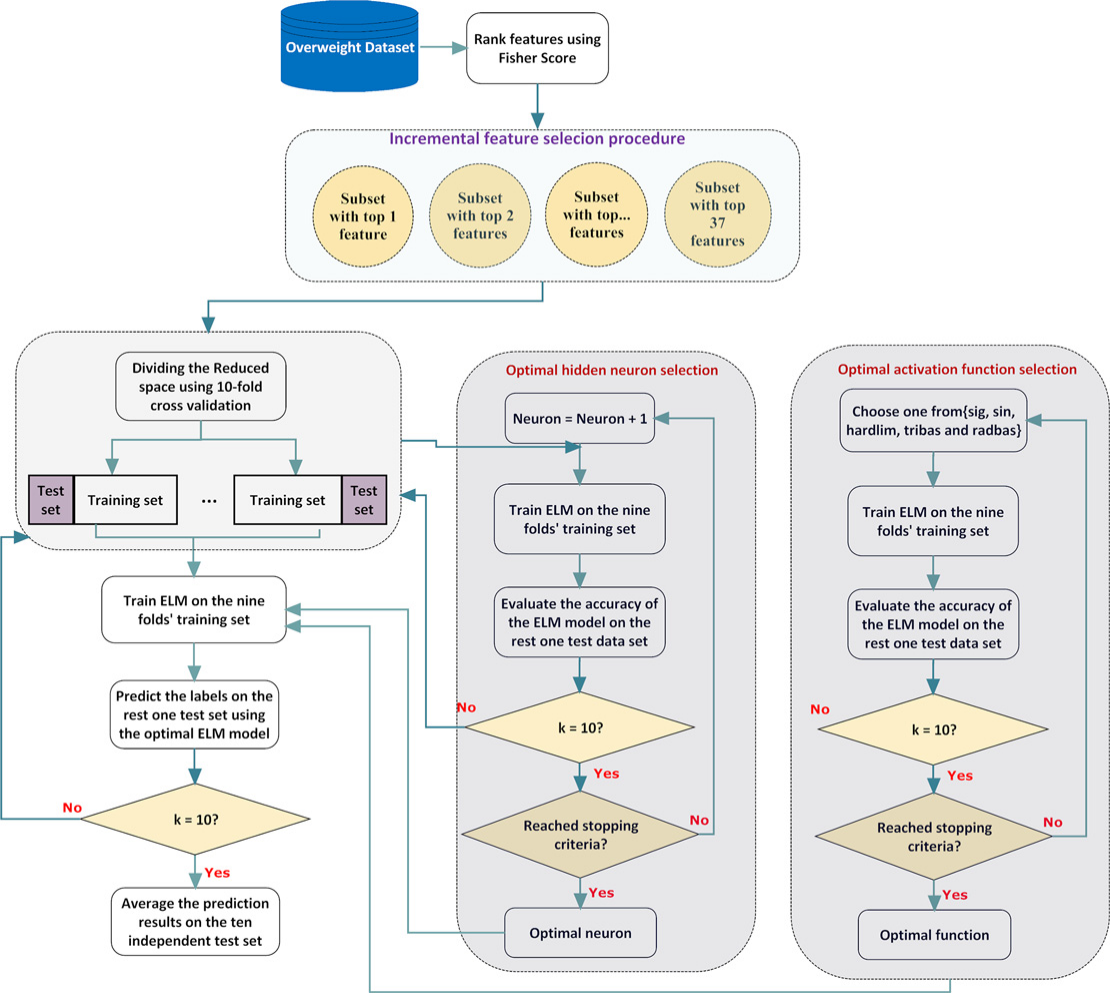
Overall procedure of the proposed method.

This section briefly describes the proposed method. The proposed method provides an efficient and accurate prediction tool for distinguishing overweight from healthy people using blood indexes. Fig 1 illustrates the flowchart of the proposed method. First, feature selection identifies the informative blood indexes. An incremental feature selection procedure is then performed to find the most representative feature subsets with top ranked features. During this procedure, features in the ranked feature list are added one-by-one from high to low rank. A total of 37 feature sets are constructed; the set that yields the best classification performance is considered the optimal feature set. Finally, the obtained optimal feature set is fed to the ELM model for performance evaluation. Before the ELM model is evaluated, it is important to first address the selection of hidden neurons and activation functions. To determine the optimal ELM structure, both issues are handled via the 10-fold cross validation (CV) analysis. The Sigmoid function (sig), Sine function (sin), Hard-limit function (hardlim), Triangular basis function (tribas) and Radial basis function (radbas) are used for evaluation. Different hidden neurons of 5, 20, 35, 50, 65, 80 and 95 are chosen for comparison. The pseudocode of the proposed method is given below.

Begin

     For *j* = 1:k

          Training set ← *k*-1 subsets;

          Validation set ← remaining subset;

          Rank features incrementally using the Fisher Score 

     *F*_*i*_ = {*f*_1_,⋯*f*_*i*_,⋯,*f*_37_}(1 ≤ *i* ≤ 37);

          Train the ELM classifier on each feature subset *f*_*i*_ with the top *i* ranked features using a variation of the number of hidden neurons and type of activation functions;

          Evaluate the trained ELM model on the validation set with the corresponding reduced feature set;

     EndFor;

          Return the average classification accuracy rates of ELM over the *j*th validation set;

End.

### 4. Experimental Designs

#### 4.1 Experimental Setup

To verify the proposed ELM approach, the state-of-the-art SVM and the commonly used ANN method in overweight modeling were employed for comparison. The famous back propagation neural network (BPNN) with the Levenberg-Marquardt training algorithm in the MATALAB neural network toolbox was adopted. Implementation code available at http://www3.ntu.edu.sg/home/egbhuang was used to construct the ELM model. For SVM, the LIBSVM toolbox developed by Chang and Lin [26] was adopted. The Fisher Score feature selection method was implemented from scratch in the MATALAB.

Data was scaled into the range [–1, 1] before classification. The empirical experiment was conducted on an AMD Athlon 64 X2 Dual Core Processor 5000+ (2.6 GHz) with 4GB of RAM running Windows 7.

#### 4.2 Data Division

The k-fold CV [27] was used to evaluate classification performance to guarantee unbiased results. The value of k is often set to 10 in the literature. As a result, whole data samples will be randomly split into 10 subsets; each time, nine subsets are used for training and the remaining one is used as the test set. The process ran 10 times. The final result was computed by averaging the result across all 10 trials. It should be noted that it is more reasonable to keep the same proportion of the samples in each fold as that of the entire dataset when splitting the data; therefore, the above stratified k-fold CV strategy is employed for analysis in the following experiment.

#### 4.3 Evaluation Criteria

To evaluate the proposed method, commonly used evaluation criteria such as classification accuracy (ACC), the area under the receiver operating characteristic curve (AUC) [28], sensitivity, and specificity were analyzed. They are defined as follows:

$$ACC=\frac{TP+TN}{TP+FP+FN+TN}\times 100\%$$ (6)

$$Sensitivity=\frac{TP}{TP+FN}\times 100\%$$ (7)

$$Specificity=\frac{TN}{FP+TN}\times 100\%$$ (8)

where TP, FN, TN, and FP are the number of true positives, false negatives, true negatives and false positives, respectively. AUC is one of the most popular methods for evaluating the performance of the binary classifier. A perfect classifier provides an AUC of 1. This study adopted the AUC algorithm developed in [29].

## Results

### 1. ELM Classification Performance

**Fig 2:**
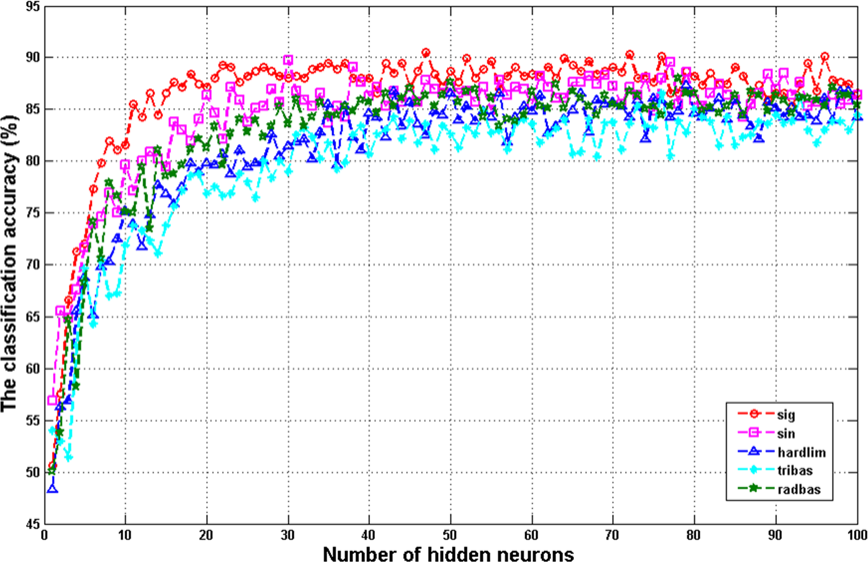
The relationship between ELM with different activation functions and the different number of hidden neurons.

Previous studies [14, 30] showed that the activation functions and hidden neurons have more or less impact on ELM performance. Therefore, these two factors were investigated in the following experiment. The influence of different activation functions on the performance of the ELM model was investigated. Five activation functions including sig, sin, hardlim, tribas, and radbas were used. Fig 2 displays the classification accuracy of ELM with different activation functions from the function of the different number of neurons. The ELM with the sig activation function outperforms ELM with other functions. Therefore, the Sigmoid function was employed in subsequent experiment analysis.

**Fig 3:**
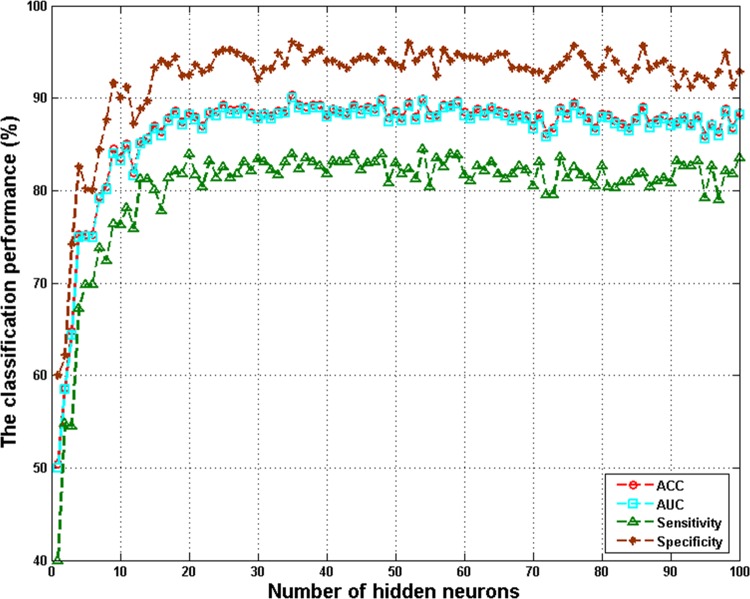
ACC, AUC, sensitivity, and specificity versus different hidden neuron numbers for ELM via 10-fold CV.

To determine the optimal number of hidden neurons, the validation accuracy as the function of the number of hidden neurons was recorded. As shown in Fig 3, the performance of ELM is relatively stable with the increase of hidden neurons. It is therefore necessary to determine the most suitable number of hidden neurons for ELM. Therefore, different models were built with different given hidden neurons of 5, 20, 35, 50, 65, 80 and 95. Table 3 presents the average classification performance results of 10-fold CV with different numbers of hidden neurons. As can be seen from the table, the classification performance of ELM models varied with differing numbers of hidden neurons. 35 hidden neurons achieved the highest validation accuracy. Therefore, 35 hidden neurons were chosen to create the training model in the following analysis. After the activation function and the number of hidden neurons were determined, the final model trained for prediction. The random input weights and the hidden layer biases acquired in this study are listed in Information S1 Table. Table 4 displays the detailed results of the 10-fold CV of the ELM. From the table, it can be seen that the ELM model achieves high performance with average results of 90.32% ACC, 89.98% AUC, 83.95% sensitivity, and 96.02% specificity.

**Table 3:** Average classification performance results of 10-fold CV.

Different number of hidden neurons	Average classification performance			
	ACC (%)	AUC (%)	Sensitivity (%)	Specificity (%)
5	76.69	76.28	68.85	83.71
20	88.44	88.02	80.81	95.23
35	**90.32**	**89.98**	**83.95**	**96.02**
50	88.87	88.56	82.71	94.42
65	87.61	87.28	81.74	92.83
80	87.39	87.10	82.15	92.05
95	87.80	87.54	82.25	92.83

The best results have been shown in bold.

**Table 4:** The detailed results obtained by the ELM model.

Fold	ACC (%)	AUC (%)	Sensitivity (%)	Specificity (%)
1#	89.36	88.91	81.82	96.00
2#	91.67	91.48	86.96	96.00
3#	87.50	87.30	82.61	92.00
4#	91.67	91.48	86.96	96.00
5#	91.67	91.30	82.61	100.00
6#	93.75	93.65	91.30	96.00
7#	91.67	91.26	86.36	96.15
8#	87.23	86.36	72.73	100.00
9#	89.36	89.18	86.36	92.00
10#	89.36	88.91	81.82	96.00
Avg.	90.32	89.98	83.95	96.02
Dev.	2.09	2.23	4.97	2.67

### 2. Comparison with SVM and BPNN

To verify the effectiveness of the ELM model, the SVM with RBF kernel and BPNN were implemented for comparison in the whole feature space on the same dataset. For SVM, a grid-search technique [31] was employed using 10-fold CV to determine the optimal parameter values of the RBF kernel function. The range of related parameters C and γ varied between C = {2^−5^, 2^−3^,…,2^15^} and γ = {2^−15^,2^−13^,…,2^1^}. 99 parameter combinations of (C, γ) were tried (Training accuracy surface of SVM with parameters obtained by grid search is listed in Information S1 Fig); the one with the best CV accuracy was chosen as the parameter value of the RBF kernel. Then, the best (C, γ) parameter pair was used to create the training model. Concerning BPNN, the three layer BP network was used, and different settings of the number of nodes in the hidden layers (5, 10, 15, 20, 25 and 30) and the different learning epochs (50, 100, 200 and 300) were tried as training stopping criteria. According to the preliminary simulation results, the best result was obtained with the hidden nodes of 10 and the learning epoch of 200. These parameter settings were used for the subsequent analysis.

**Fig 4:**
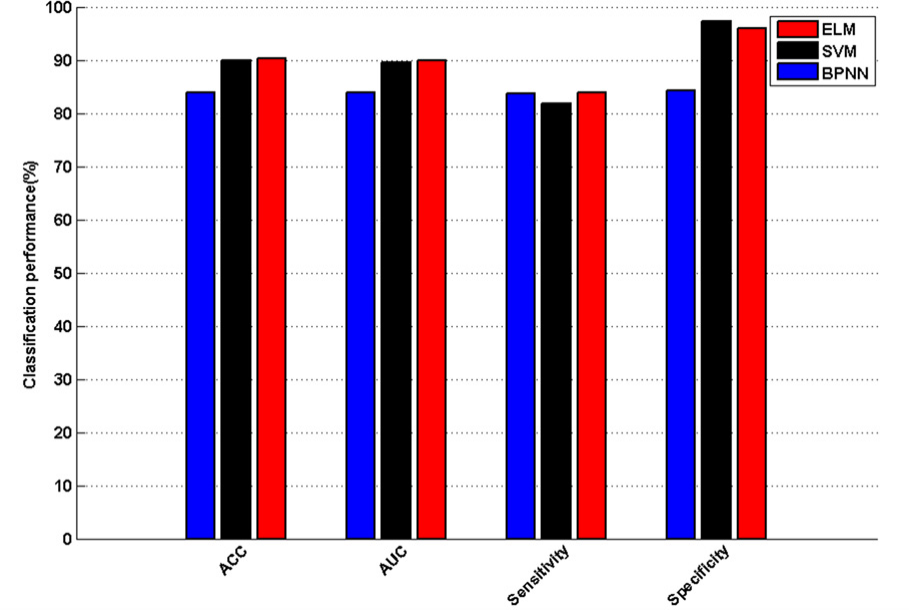
Comparison results of classification performance among ELM, SVM, and BPNN.

Fig 4 compares the ACC, AUC, sensitivity, and specificity of ELM, SVM, and BPNN. ELM performed the best among the three methods in terms of ACC, AUC, and sensitivity. Compared to ELM, SVM achieved slightly higher specificity, and BPNN obtained similar sensitivity. Table 5 also presents the confusion matrices obtained by the three methods via 10-fold CV analysis. As can be seen from Table 5, ELM correctly recognized 189 out of 225 overweight cases and 241 out of 251 healthy cases. It misclassified 36 overweight cases as healthy cases and 10 healthy cases as overweight cases. SVM correctly classified 184 out of 225 overweight cases and 244 out of 251 healthy cases. It misclassified 41 overweight cases and 7 healthy cases. BPNN correctly recognize 188 out of 225 overweight cases and 211 out of 251 healthy cases. It misclassified 37 overweight cases and 40 healthy cases. Based on these results, ELM and SVM outperformed BPNN, especially concerning the capability to recognize healthy cases. Additionally, ELM is more effective than SVM and BPNN at discriminating overweight from healthy controls. These results verify that ELM has better generalization capabilities than other counterparts. The CPU time needed for the training procedure of ELM, SVM, and BPNN were also recorded in the experiment. SVM required 95 seconds, BPNN required 169 seconds, and ELM required 0.5 seconds. ELM is far more efficient than SVM and BPNN for overweight recognition.

**Table 5:** The confusion matrix obtained by ELM, SVM and BPNN via 10-fold CV.

ELM	Predicted overweight people	Predicted healthy controls
Overweight people	189	36
Healthy controls	10	241
**SVM**		
Overweight people	184	41
Healthy controls	7	244
**BPNN**		
Overweight people	188	37
Healthy controls	40	211

### 3. Classification Results Based on Feature Selection

**Fig 5:**
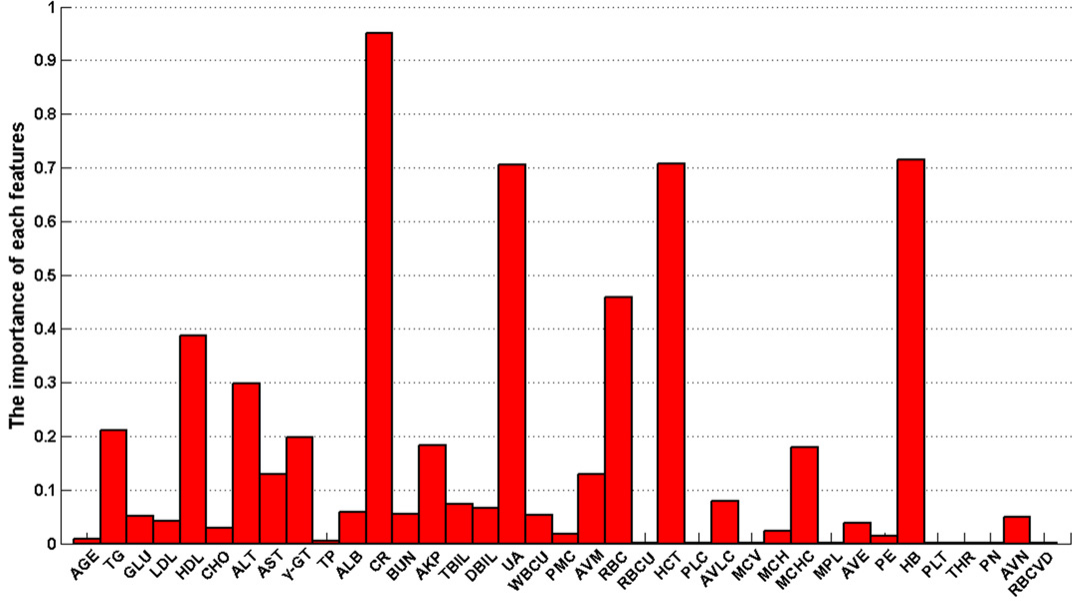
The importance of each index obtained by the Fisher Score feature selection.

**Fig 6:**
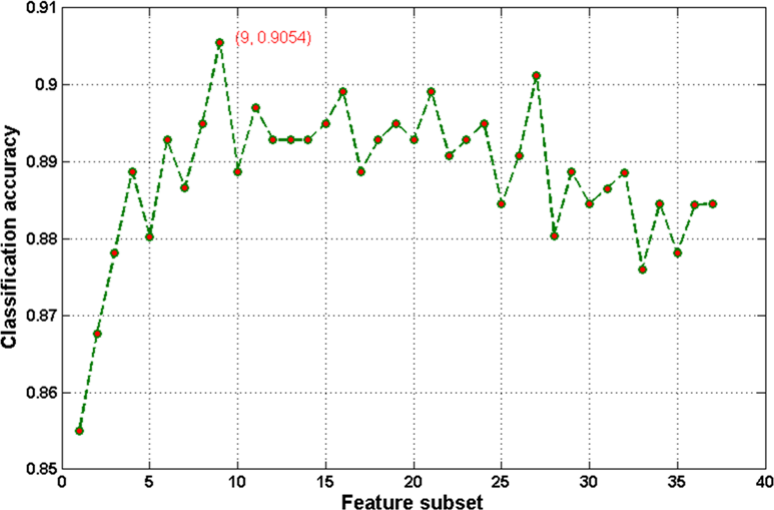
The incremental feature selection curve: the classification accuracy against the feature subset.

**Fig 7:**
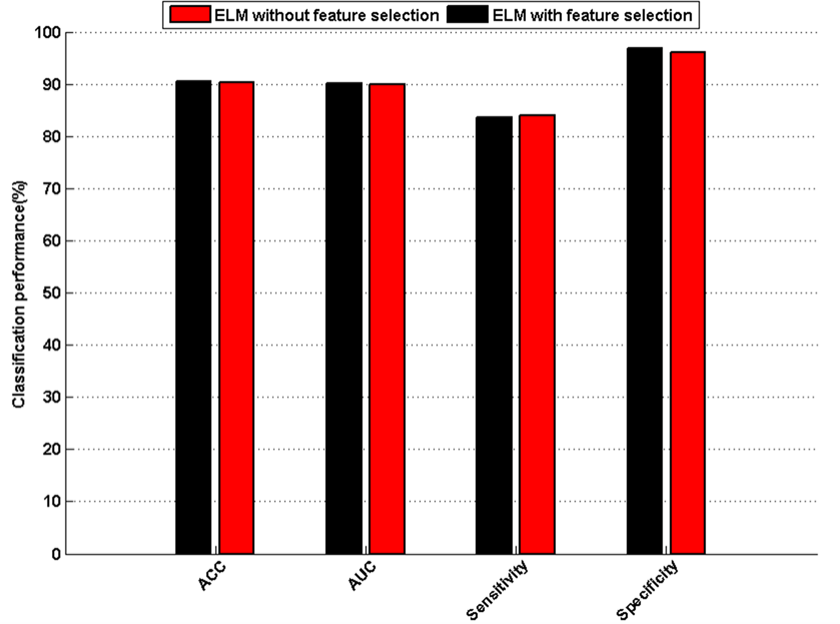
Comparison results of classification performance between ELM with and without feature selection.

To identify the most relevant factors determining the overweight status, feature selection was implemented to rank features before classification. Based on the Fisher Score feature selection algorithm, a ranked feature list was obtained on the basis of each feature’s relevance to the sample class. As shown in Fig 5, the importance of each index is computed; these lists of ranked features were used to establish the optimal feature subset in the incremental feature selection procedure. 37 different feature subsets can be obtained by adding features one-by-one from higher to lower rank according to importance. The ELM classifier is accordingly built for each feature subset and incrementally evaluated by 10-fold CV. Fig 6 plots the incremental feature selection curve, which reveals the relationship between the classification accuracy and the feature subset. The classification performance obtained on the top ranked five, seven and nine features is shown in Information S2 Table. The maximal classification accuracy is obtained at 90.54% when the top nine ranked features comprise the feature set. These nine features corresponded to the nine blood indexes of CR, HB, HCT, UA, RBC, HDL, ALT, TG and γ-GT. These nine features were selected as the optimal feature set to construct the classifier. The hierarchical clustering heat map is developed based on the nine selected features as shown in Information S2 Fig. The optimal feature set results in an average of 90.54% ACC, 90.17% AUC, 83.54% sensitivity, and 96.80% specificity for ELM. Fig 7 shows the comparison results from ELM without feature selection versus ELM with the optimal feature set in terms of ACC, AUC, sensitivity, and specificity via 10-fold CV analysis. The ACC, AUC, and specificity of the optimal feature set are all superior to those of the whole feature set. The sensitivity of the optimal feature set is also comparable to that of the original feature set. Table 6 lists the results of the ELM confusion matrix on the whole feature set and the optimal feature set. ELM with the optimal feature set and ELM with the original feature set had almost the same capability to discriminate overweight cases from the healthy controls. These results suggest that the original feature set contained redundant or irrelevant information. It also indicates that the Fisher Score feature selection has the capability to identify the most informative features.

**Table 6:** The confusion matrix obtained by ELM with and without feature selection.

ELM with the whole feature set	Predicted overweight people	Predicted healthy controls
Overweight people	189	36
Healthy controls	10	241
**ELM with the optimal feature set**		
Overweight people	188	37
Healthy controls	8	243

## Discussion

Routine blood tests include evaluations of liver function, renal function, and blood-lipid and glucose levels. This data can identify the physiological state of a subject. Overweight subjects have different metabolic activity compared to healthy subjects, resulting in detectable differences. A clinical study on a teenage population to study obesity-related alterations in laboratory parameters confirmed that obese subjects have systematical changes in blood test parameters [32]. Obesity has been considered a systematic, low-level, chronic inflammation state that is heritable and predisposes the subject to many diseases [33]. Clearly detecting the overweight status could have great clinical significance.

In this study, routine blood tests were obtained and One-Way ANOVA analysis showed that there are significant differences between overweight and healthy subjects (p-value < 0.01) in their TG, GLU, LDL, HDL, CHO, ALT, AST, γ-GT, ALB, CR, BUN, AKP, TBIL, DBIL, UA, WBCU, PMC, AVM, RBC, HCT, AVLC, MCH, MCHC, AVE, PE, HB, AVN. Of these, TG, LDL, HDL and CHO belong to blood-lipid test indexes; ALT, AST, γ-GT, ALB, AKP, TBIL and DBIL belong to liver functions; CR, BUN and UA belong to renal functions; WBCU, PMC, AVM, RBC, HCT, AVLC, MCH, MCHC, AVE, PE, HB and AVN belong to blood routine tests. However, ANOVA analysis can only show differences, not correlation.

According to the results of the feature selection, the most important indexes that correlate with the overweight status are CR, HB, HCT, UA, RBC, HDL, ALT, TG and γ-GT, which is consistent with the results of the Spearman test analysis. These indexes have statistically significant differences (p-value < 0.01). Among these blood parameters, CR and UA are renal function indexes. CR is a nitrogenous metabolic substance in muscles and blood. Uric acid is produced from the natural breakdown of cells and foods. Overweight subjects have fast metabolisms; they consume more calories per day than healthy subjects. Therefore, it is understandable that CR and UA were identified as two main features. HB is a protein in red blood cells; HCT is the volume percentage of red blood cells; RBC (erythrocytes) takes oxygen from the lungs and releases it into tissues. These are the three most important and relevant indexes for delivering oxygen. As overweight person consumes more calories and therefore requires more oxygen; this is consistent with elevated CR and UA. ALT and γ-GT are found mainly in the liver and are reliable indexes for liver damage. The liver is the metabolic factory in the human body; therefore, the obtained results in this study are reliable and have medical significance. The physiological state was different for overweight subjects; the renal function state, particularly the CR level, is a correlated parameter.

In this study, overweight and healthy subjects were classified according to BMI, which correlated with body fat. Body fat is the most common index used to assess the overweight status [34]. It also serves as a biomarker in hyperuricemia [35], and can predict the severity of coronary artery disease [36], nonalcoholic fatty liver disease [37], and the clustering of cardiometabolic risk factors in adolescents [38]. When BMI is measured at the base line, it was as effective as 2-h plasma glucose and fasting plasma glucose to predict diabetes [39]. Although BMI has been widely used as a diagnosis criterion for overweight subjects, it is not an accurate predicting indicator for some diseases. For instance, in middle-aged and older women, waist circumference is a more sensitive measurement of relative disease risk than is BMI [40]. The waist-to-height ratio showed better correlation with the severity of coronary artery disease than BMI [36]. Few studies examined in the relationship between BMI and blood biochemical changes in overweight subjects. Based on this study, selected blood indexes including HB, HCT, UA, RBC, HDL, ALT, TG and γ-GT can be used with BMI to assess the overweight status or predict some obesity-related diseases. Combined with the results of our findings, BMI will be a useful tool in the clinical practice.

## Conclusion

This study attempted to determine an effective data-driven machine learning model for discriminating overweight from healthy controls using blood and biochemical indexes for the first time. In addition, correlating factors from the blood parameters were also investigated in overweight subjects by introducing the feature selection technique. A new machine learning paradigm, ELM, was explored and the classification performance was compared against the competitive SVM and BPNN in various evaluation metrics. Experimental results show that the ELM performs much more efficiently than the SVM and BPNN, and with higher recognition rates. Nine informative indexes that strongly correlate the overweight condition were identified with the feature selection; they include CR, HB, HCT, UA, RBC, HDL, ALT, TG and γ-GT.

Based on these discoveries, the proposed ELM-based approach for overweight detection in biomedical applications has promising potential. It provides a viable alternative solution to traditional overweight modeling tools by offering excellent predictive ability. However, it should be noted that only 225 overweight people were involved in the experiment; more data samples are needed to further verify the effectiveness of the proposed methodology.

## Supporting Information

S1 Fig Training accuracy surface of SVM with parameters obtained by grid search. The file lists the training accuracy surface of SVM with parameters obtained by grid search.
(TIF)

S2 Fig The hierarchical clustering heat map developed on the nine selected features. The file lists the hierarchical clustering heat map developed on the nine selected features.
(TIF)

S1 Table The random biases and input weights. The file lists the randomly produced biases and input weights produced by the ELM during training.
(XLSX)

S2 Table The classification performance of ELM obtained on the top ranked five, seven and nine features. The file lists the classification performance of ELM obtained on the top ranked five, seven and nine features.
(DOCX)
