# Supplementary figures and images for: Using Blood Indexes to Predict Overweight Statuses: An Extreme Learning Machine-Based Approach
Source: PLoS One. 2015 Nov 23;10(11):e0143003. doi: 10.1371/journal.pone.0143003 (PMC4658146; doi:10.1371/journal.pone.0143003)

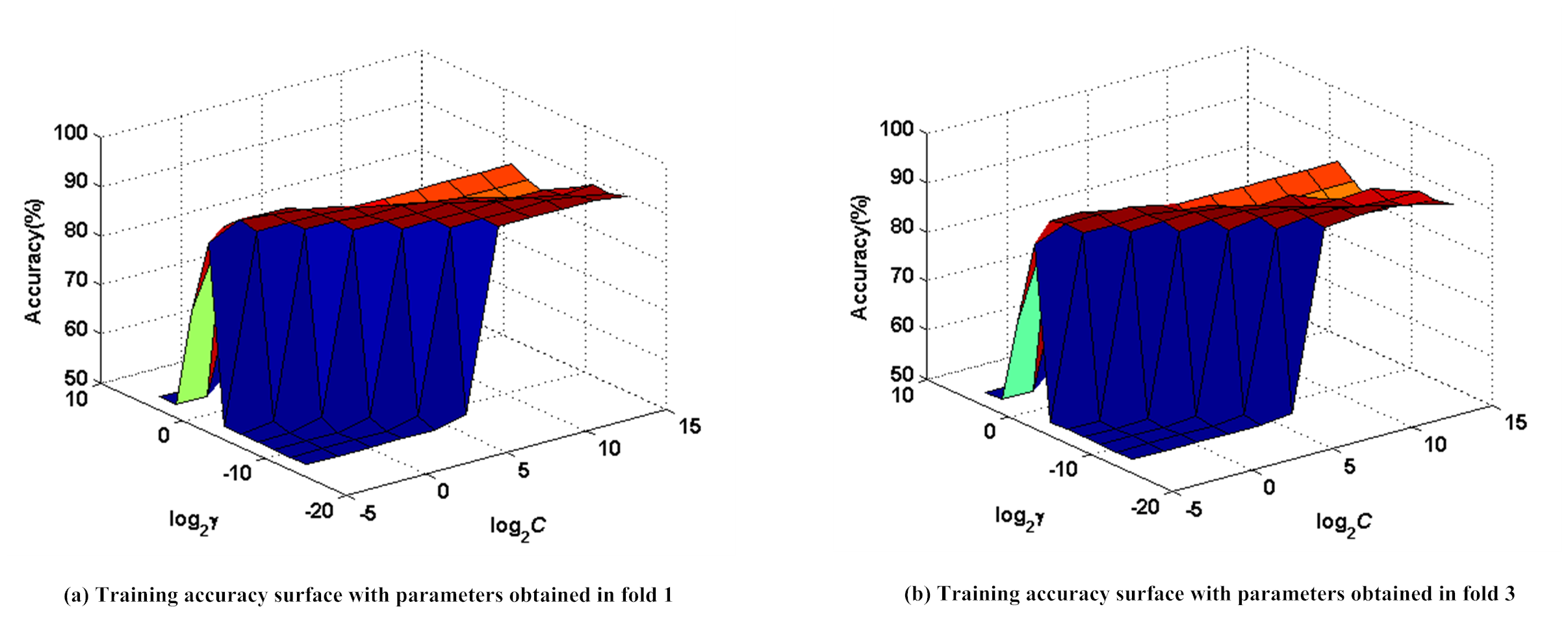

Supplement: S1 Fig — The file lists the training accuracy surface of SVM with parameters obtained by grid search. (TIF) [file pone.0143003.s001.tif]

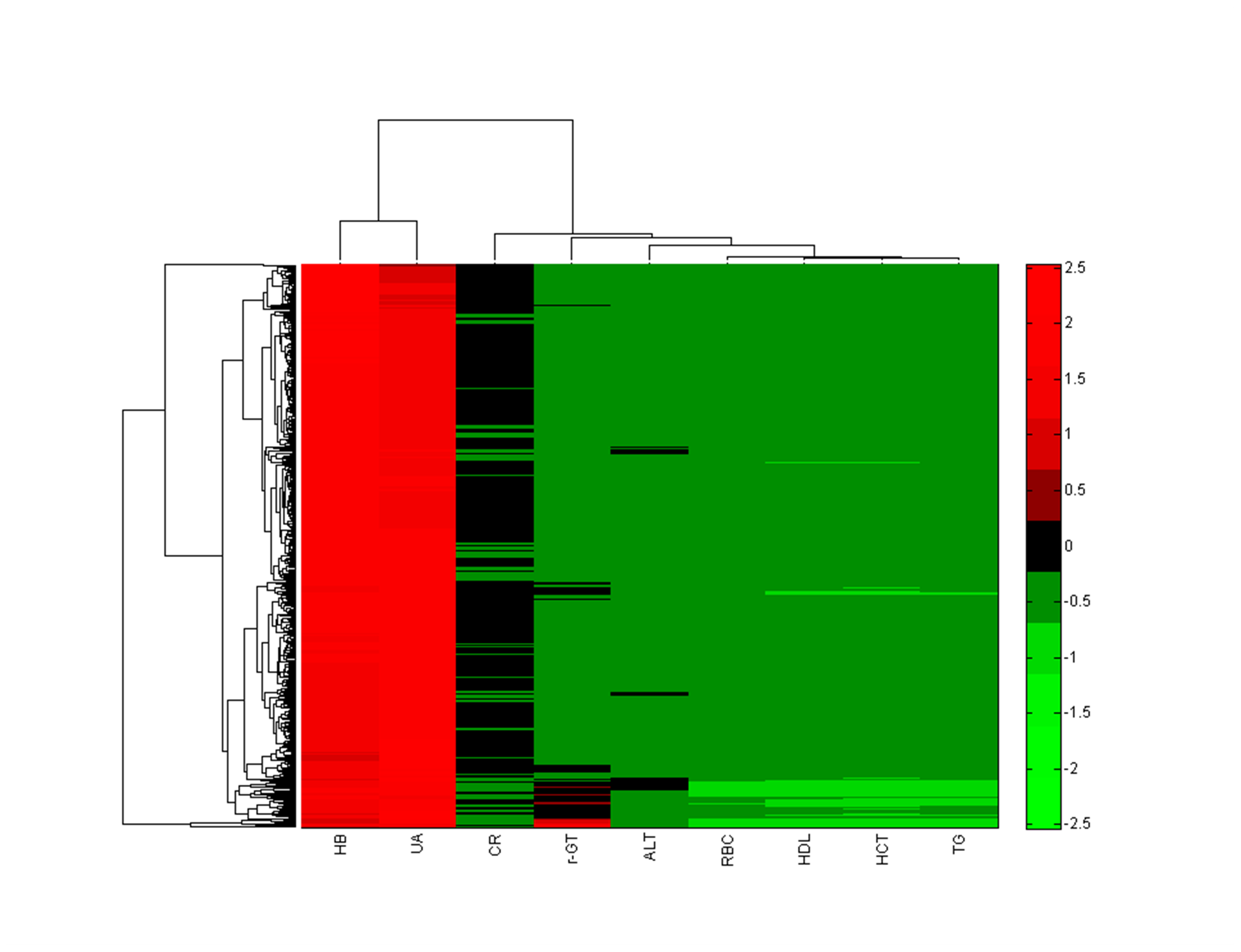

Supplement: S2 Fig — The file lists the hierarchical clustering heat map developed on the nine selected features. (TIF) [file pone.0143003.s002.tif]
