# Supplementary material for: Using Blood Indexes to Predict Overweight Statuses: An Extreme Learning Machine-Based Approach
Source: PLoS One. 2015 Nov 23;10(11):e0143003. doi: 10.1371/journal.pone.0143003 (PMC4658146; doi:10.1371/journal.pone.0143003)
Supplement: S2 Table — The file lists the classification performance of ELM obtained on the top ranked five, seven and nine features. (DOCX) [file pone.0143003.s004.docx]

**Table 1. The average performance obtained on the different number of top features.**

| Different number of top features | Average classification performance | | | |
| --- | --- | --- | --- | --- |
|  | ACC (%) | AUC (%) | Sensitivity (%) | Specificity (%) |
| 5 | 88.22 | 87.88 | 82.15 | 93.62 |
| 7 | 89.68 | 89.35 | 83.50 | 95.20 |
| 9 | 90.54 | 90.17 | 83.54 | 96.80 |
